# Crystal structure of 5-chloro-3-cyclo­hexyl­sulfinyl-2,4,6-trimethyl-1-benzo­furan

**DOI:** 10.1107/S1600536814019217

**Published:** 2014-08-30

**Authors:** Hong Dae Choi, Uk Lee

**Affiliations:** a Department of Chemistry, Dongeui University, San 24 Kaya-dong, Busanjin-gu, Busan 614-714, Republic of Korea; b Department of Chemistry, Pukyong National University, 599-1 Daeyeon 3-dong, Nam-gu, Busan 608-737, Republic of Korea

**Keywords:** crystal structure, benzo­furan, cyclo­hex­yl, C—H⋯O hydrogen bonds, C—H⋯π inter­actions

## Abstract

In the title compound, C_17_H_21_ClO_2_S, the cyclo­hexyl ring adopts a chair conformation with the C—S bond in an equatorial orientation. In the crystal, mol­ecules are linked by C—H⋯O and C—H⋯π hydrogen bonds and a Cl⋯π [3.594 (2) Å] contact into chains along the *a*-axis direction.

## Related literature   

For the biological activity of benzo­furan compounds, see: Aslam *et al.* (2009 ▶); Galal *et al.* (2009 ▶); Howlett *et al.* (1999 ▶); Khan *et al.* (2005 ▶); Ono *et al.* (2002 ▶). For natural products with a benzo­furan ring, see: Akgul & Anil (2003 ▶); Soekamto *et al.* (2003 ▶). For the synthesis of the starting material 5-chloro-3-cyclo­hexyl­sulfanyl-2,4,6-trimethyl-1-benzo­furan, see: Choi *et al.* (1999 ▶). For a related structure, see: Choi *et al.* (2011 ▶).
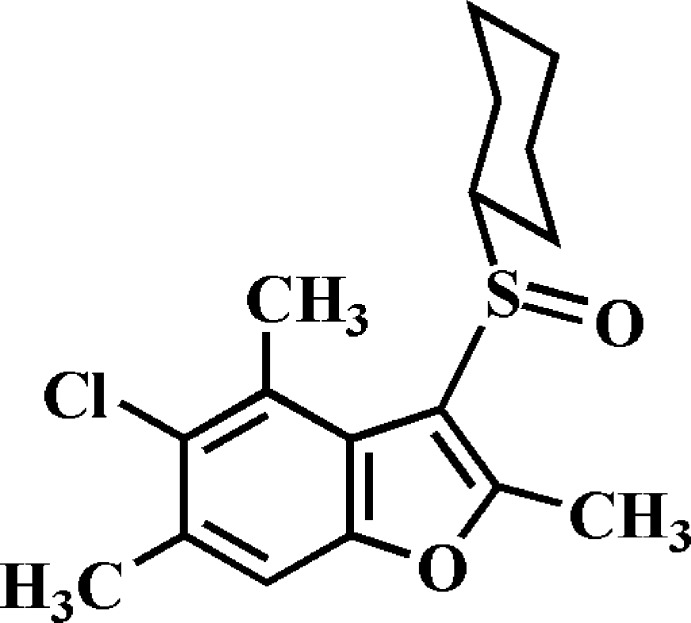


## Experimental   

### Crystal data  

C_17_H_21_ClO_2_S
*M* *_r_* = 324.85
Triclinic, 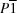

*a* = 5.8612 (1) Å
*b* = 11.6832 (2) Å
*c* = 12.6432 (2) Å
α = 65.292 (1)°
β = 85.902 (1)°
γ = 83.229 (1)°
*V* = 780.79 (2) Å^3^
*Z* = 2
Mo *K*α radiation
μ = 0.38 mm^−1^
*T* = 173 K
0.31 × 0.24 × 0.23 mm

### Data collection  

Bruker SMART APEXII CCD diffractometer
Absorption correction: multi-scan (*SADABS*; Bruker, 2009 ▶) *T*_min_ = 0.892, *T*_max_ = 0.917
13925 measured reflections
3588 independent reflections
3221 reflections with *I* > 2σ(*I*)
*R*_int_ = 0.024

### Refinement  

*R*[*F* ^2^ > 2σ(*F* ^2^)] = 0.039
*wR*(*F* ^2^) = 0.102
*S* = 1.03
3588 reflections
193 parameters
H-atom parameters constrained
Δρ_max_ = 0.77 e Å^−3^
Δρ_min_ = −0.37 e Å^−3^

### 

Data collection: *APEX2* (Bruker, 2009 ▶); cell refinement: *SAINT* (Bruker, 2009 ▶); data reduction: *SAINT*; program(s) used to solve structure: *SHELXS97* (Sheldrick, 2008 ▶); program(s) used to refine structure: *SHELXL97* (Sheldrick, 2008 ▶); molecular graphics: *ORTEP-3 for Windows* (Farrugia, 2012 ▶) and *DIAMOND* (Brandenburg, 1998 ▶); software used to prepare material for publication: *SHELXL97*.

## Supplementary Material

Crystal structure: contains datablock(s) I. DOI: 10.1107/S1600536814019217/hb7277sup1.cif

Structure factors: contains datablock(s) I. DOI: 10.1107/S1600536814019217/hb7277Isup2.hkl

Supporting information file. DOI: 10.1107/S1600536814019217/hb7277Isup3.cml

**Fig. 1.**
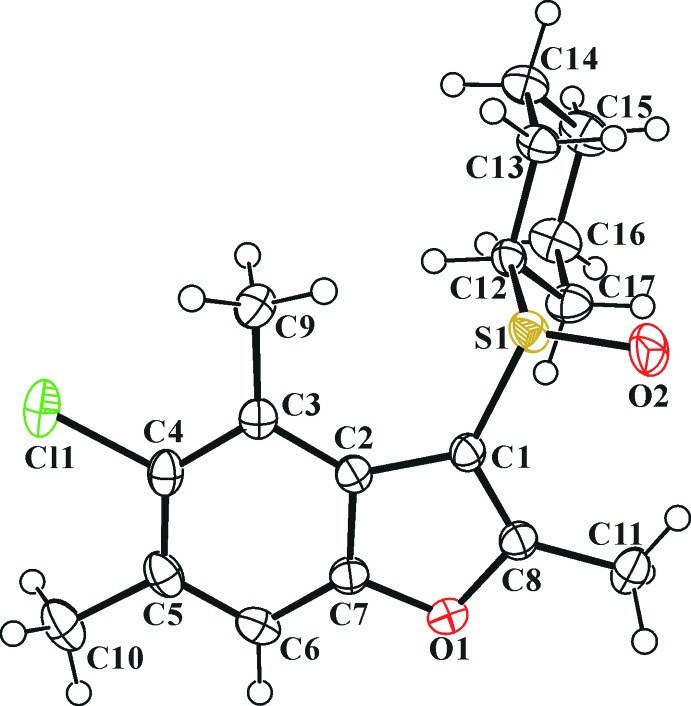
The molecular structure of the title compound with displacement ellipsoids drawn at the 50% probability level.

**Fig. 2.**
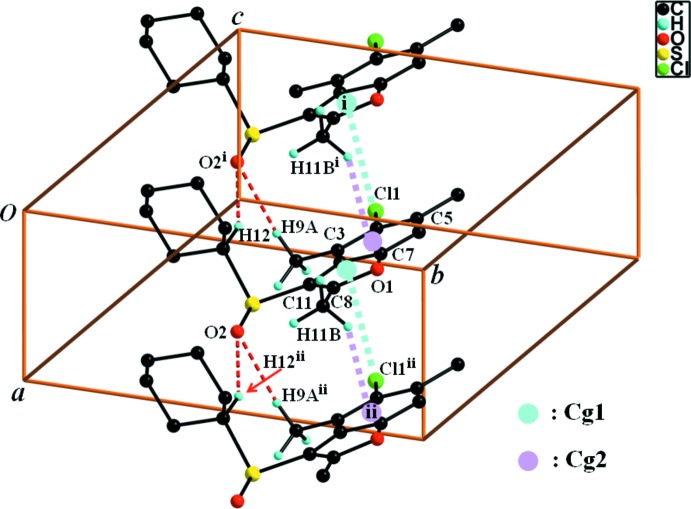
A view of the C—H···O, C—H···π and C—Cl···π interactions (dotted lines) in the crystal structure of the title compound. H atoms non-participating in hydrogen-bonding were omitted for clarity. [Symmetry codes: (i) x - 1, y, z; (ii) x + 1, y, z.]

. DOI: 10.1107/S1600536814019217/hb7277fig1.tif
The mol­ecular structure of the title compound with displacement ellipsoids drawn at the 50% probability level.

x y z x y z . DOI: 10.1107/S1600536814019217/hb7277fig2.tif
A view of the C—H⋯O, C—H⋯π and C—Cl⋯π inter­actions (dotted lines) in the crystal structure of the title compound. H atoms non-participating in hydrogen-bonding were omitted for clarity. [Symmetry codes: (i) *x* − 1, *y*, *z*; (ii) *x* + 1, *y*, *z*.]

CCDC reference: 1021106

Additional supporting information:  crystallographic information; 3D view; checkCIF report

## Figures and Tables

**Table 1:** Hydrogen-bond geometry (Å, °) *Cg*2 is the centroid of the C2–C7 benzene ring.

*D*—H⋯*A*	*D*—H	H⋯*A*	*D*⋯*A*	*D*—H⋯*A*
C9—H9*A*⋯O2^i^	0.98	2.53	3.438 (2)	154
C12—H12⋯O2^i^	1.00	2.39	3.3072 (19)	152
C11—H11b⋯*Cg*2^i^	0.98	2.83	3.533 (2)	129

Symmetry code: (i) 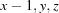.

## supplementary crystallographic information

### S1. Comment

Benzofuran compounds show significant pharmacological properties such as antibacterial and antifungal, antitumor and antiviral, antimicrobial activities (Aslam *et al.* 2009, Galal *et al.*, 2009, Khan *et al.* , 2005), and inhibitor of β-amyloid aggregation (Howlett *et al.*, 1999, Ono *et al.*, 2002). These many benzofurans occur in a great number of natural products (Akgul & Anil, 2003, Soekamto *et al.*, 2003). As a part of our ongoing project of 5-chloro-3-cyclohexylsulfinyl-1-benzofuran derivatives containing methyl substituent in 2-position (Choi *et al.*, 2011), we report herein on the crystal structure of the title compound.

In the title molecule (Fig. 1), the benzofuran unit is essentially planar, with a mean deviation of 0.014 (1) Å from the least-squares plane defined by the nine constituent atoms. The cyclohexyl ring is in the chair form and the arylsulfinyl moiety is positioned equatorially relative to the cyclohexyl group. In the crystal structure (Fig. 2), molecules are linked by C—H···O and C—H···π hydrogen bonds (Table 1, Cg2 is the centroid of the C2–C7 benzene ring). The molecules are stacked along the *a*-axis. A Cl···π contact between the chlorine atom and the furan ring of an adjacent molecule, with Cl1···Cg1^i^ [3.594 (2) Å] (Cg1 is the centroid of the C1/C2/C7/O1/C8 furan ring) is observed, compared to the van der Waals' separation of 3.55Å for these species.

### S2. Experimental

The starting material 5-chloro-3-cyclohexylsulfanyl-2,4,6-trimethyl-1-benzofuran was prepared by literature method (Choi *et al.* 1999). 3-Chloroperoxybenzoic acid (77%, 224 mg, 1.0 mmol) was added in small portions to a stirred solution of 5-chloro-3-cyclohexylsulfanyl-2,4,6-trimethyl-1-benzofuran (278 mg, 0.9 mmol) in dichloromethane (20 mL) at 273 K. After being stirred at room temperature for 5h, the mixture was washed with saturated sodium bicarbonate solution (2 × 10 ml) and the organic layer was separated, dried over magnesium sulfate, filtered and concentrated at reduced pressure. The residue was purified by column chromatography (hexane–ethyl acetate, 2:1 *v*/*v*) to afford the title compound as a colorless solid [yield 77% (250 mg); m.p. 449–450 K; *R*_f_ = 0.61 (hexane–ethyl acetate, 2:1 *v*/*v*)]. Colourless blocks were prepared by slow evaporation of a solution of the title compound (26 mg) in ethyl acetate (10 ml) at room temperature.

### S3. Refinement

All H atoms were positioned geometrically and refined using a riding model, with C—H = 0.95 Å for aryl, 1.00 Åfor methine, 0.99 Å for methylene and 0.98 Å for methyl H atoms, respectively. Uiso = 1.2Ueq (C) for aryl, methine and methylene, and 1.5Ueq for methyl H atoms. The positions of methyl and methylene hydrogens were optimized using the SHELXL-97 command AFIX 137 (Sheldrick, 2008).

### Crystal data

**Table:**

C_17_H_21_ClO_2_S	*Z* = 2
*M**_r_* = 324.85	*F*(000) = 344
Triclinic, *P*1	*D*_x_ = 1.382 Mg m^−^^3^
Hall symbol: -P 1	Melting point = 417–416 K
*a* = 5.8612 (1) Å	Mo *K*α radiation, λ = 0.71073 Å
*b* = 11.6832 (2) Å	Cell parameters from 5736 reflections
*c* = 12.6432 (2) Å	θ = 3.1–27.5°
α = 65.292 (1)°	µ = 0.38 mm^−^^1^
β = 85.902 (1)°	*T* = 173 K
γ = 83.229 (1)°	Block, colourless
*V* = 780.79 (2) Å^3^	0.31 × 0.24 × 0.23 mm

### Data collection

**Table:**

Bruker SMART APEXII CCD diffractometer	3588 independent reflections
Radiation source: rotating anode	3221 reflections with *I* > 2σ(*I*)
Graphite multilayer monochromator	*R*_int_ = 0.024
Detector resolution: 10.0 pixels mm^-1^	θ_max_ = 27.5°, θ_min_ = 1.8°
φ and ω scans	*h* = −7→6
Absorption correction: multi-scan (*SADABS*; Bruker, 2009)	*k* = −15→15
*T*_min_ = 0.892, *T*_max_ = 0.917	*l* = −16→16
13925 measured reflections	

### Refinement

**Table:**

Refinement on *F*^2^	Primary atom site location: structure-invariant direct methods
Least-squares matrix: full	Secondary atom site location: difference Fourier map
*R*[*F*^2^ > 2σ(*F*^2^)] = 0.039	Hydrogen site location: difference Fourier map
*wR*(*F*^2^) = 0.102	H-atom parameters constrained
*S* = 1.03	*w* = 1/[σ^2^(*F*_o_^2^) + (0.0521*P*)^2^ + 0.403*P*] where *P* = (*F*_o_^2^ + 2*F*_c_^2^)/3
3588 reflections	(Δ/σ)_max_ = 0.001
193 parameters	Δρ_max_ = 0.77 e Å^−^^3^
0 restraints	Δρ_min_ = −0.37 e Å^−^^3^

### Special details

**Table:**

Experimental. ^1^H NMR (δ p.p.m., CDCl3, 400 Hz): 7.19 (s, 1H), 2.73 (s, 3H), 2.36 (s, 3H), 2.31 (s, 3H), 1.63-2.12 (m, 5H), 1.10-1.58 (m, 6H).
Geometry. All esds (except the esd in the dihedral angle between two l.s. planes) are estimated using the full covariance matrix. The cell esds are taken into account individually in the estimation of esds in distances, angles and torsion angles; correlations between esds in cell parameters are only used when they are defined by crystal symmetry. An approximate (isotropic) treatment of cell esds is used for estimating esds involving l.s. planes.
Refinement. Refinement of F^2^ against ALL reflections. The weighted R-factor wR and goodness of fit S are based on F^2^, conventional R-factors R are based on F, with F set to zero for negative F^2^. The threshold expression of F^2^ > 2sigma(F^2^) is used only for calculating R-factors(gt) etc. and is not relevant to the choice of reflections for refinement. R-factors based on F^2^ are statistically about twice as large as those based on F, and R- factors based on ALL data will be even larger.

### Fractional atomic coordinates and isotropic or equivalent isotropic displacement parameters (Å^2^)

**Table:**

	*x*	*y*	*z*	*U*_iso_*/*U*_eq_	
Cl1	−0.27225 (7)	0.86676 (4)	0.02495 (4)	0.03675 (13)	
S1	0.57459 (7)	0.47693 (4)	0.17641 (4)	0.02576 (12)	
O1	0.47568 (19)	0.69596 (10)	0.34877 (10)	0.0266 (2)	
O2	0.8052 (2)	0.41466 (12)	0.22085 (12)	0.0382 (3)	
C1	0.4841 (3)	0.58674 (14)	0.23789 (13)	0.0218 (3)	
C2	0.2864 (2)	0.68140 (14)	0.20410 (13)	0.0213 (3)	
C3	0.1136 (3)	0.71923 (14)	0.12166 (13)	0.0225 (3)	
C4	−0.0488 (3)	0.81617 (15)	0.12320 (14)	0.0257 (3)	
C5	−0.0466 (3)	0.87677 (14)	0.19854 (15)	0.0282 (3)	
C6	0.1294 (3)	0.83926 (15)	0.27712 (15)	0.0277 (3)	
H6	0.1388	0.8783	0.3290	0.033*	
C7	0.2904 (3)	0.74323 (14)	0.27712 (13)	0.0238 (3)	
C8	0.5900 (3)	0.60035 (14)	0.32321 (14)	0.0243 (3)	
C9	0.1048 (3)	0.66135 (16)	0.03624 (14)	0.0283 (3)	
H9A	−0.0059	0.5977	0.0645	0.042*	
H9B	0.2574	0.6211	0.0279	0.042*	
H9C	0.0572	0.7275	−0.0395	0.042*	
C10	−0.2283 (3)	0.98104 (17)	0.19422 (18)	0.0377 (4)	
H10A	−0.1957	1.0125	0.2517	0.056*	
H10B	−0.3792	0.9481	0.2119	0.056*	
H10C	−0.2281	1.0503	0.1162	0.056*	
C11	0.7959 (3)	0.53609 (17)	0.39374 (15)	0.0318 (4)	
H11A	0.7481	0.4858	0.4744	0.048*	
H11B	0.8933	0.5996	0.3919	0.048*	
H11C	0.8826	0.4803	0.3616	0.048*	
C12	0.3682 (3)	0.36179 (14)	0.25301 (13)	0.0220 (3)	
H12	0.2101	0.4071	0.2406	0.026*	
C16	0.2432 (4)	0.19538 (17)	0.44265 (15)	0.0383 (4)	
H16A	0.2776	0.1507	0.5265	0.046*	
H16B	0.0837	0.2361	0.4357	0.046*	
C17	0.4088 (3)	0.29673 (16)	0.38293 (14)	0.0318 (4)	
H17A	0.3861	0.3601	0.4166	0.038*	
H17B	0.5691	0.2573	0.3966	0.038*	
C13	0.3866 (3)	0.26742 (15)	0.19771 (14)	0.0274 (3)	
H13A	0.3500	0.3125	0.1142	0.033*	
H13B	0.5459	0.2267	0.2036	0.033*	
C14	0.2215 (3)	0.16667 (16)	0.25851 (15)	0.0325 (4)	
H14A	0.0614	0.2066	0.2458	0.039*	
H14B	0.2415	0.1037	0.2243	0.039*	
C15	0.2635 (4)	0.10016 (16)	0.38823 (16)	0.0382 (4)	
H15A	0.4190	0.0539	0.4013	0.046*	
H15B	0.1500	0.0378	0.4260	0.046*	

### Atomic displacement parameters (Å^2^)

**Table:**

	*U*^11^	*U*^22^	*U*^33^	*U*^12^	*U*^13^	*U*^23^
Cl1	0.0255 (2)	0.0385 (2)	0.0353 (2)	0.00422 (17)	−0.00709 (17)	−0.00539 (18)
S1	0.0211 (2)	0.0256 (2)	0.0315 (2)	−0.00217 (14)	0.00493 (15)	−0.01365 (16)
O1	0.0263 (6)	0.0269 (6)	0.0297 (6)	−0.0042 (4)	−0.0037 (5)	−0.0137 (5)
O2	0.0201 (6)	0.0393 (7)	0.0566 (8)	0.0026 (5)	0.0011 (5)	−0.0231 (6)
C1	0.0180 (7)	0.0213 (7)	0.0255 (7)	−0.0026 (5)	0.0000 (5)	−0.0088 (6)
C2	0.0181 (7)	0.0201 (7)	0.0245 (7)	−0.0038 (5)	0.0022 (6)	−0.0078 (6)
C3	0.0203 (7)	0.0218 (7)	0.0225 (7)	−0.0044 (6)	0.0019 (6)	−0.0060 (6)
C4	0.0195 (7)	0.0243 (7)	0.0259 (8)	−0.0019 (6)	−0.0001 (6)	−0.0033 (6)
C5	0.0256 (8)	0.0198 (7)	0.0330 (8)	−0.0020 (6)	0.0059 (6)	−0.0057 (6)
C6	0.0308 (8)	0.0225 (7)	0.0319 (8)	−0.0057 (6)	0.0049 (7)	−0.0133 (6)
C7	0.0227 (7)	0.0226 (7)	0.0258 (7)	−0.0049 (6)	0.0002 (6)	−0.0092 (6)
C8	0.0209 (7)	0.0231 (7)	0.0283 (8)	−0.0044 (6)	0.0001 (6)	−0.0096 (6)
C9	0.0266 (8)	0.0322 (8)	0.0259 (8)	−0.0018 (6)	−0.0032 (6)	−0.0116 (7)
C10	0.0320 (9)	0.0264 (8)	0.0490 (11)	0.0030 (7)	0.0068 (8)	−0.0128 (8)
C11	0.0247 (8)	0.0352 (9)	0.0323 (9)	−0.0022 (7)	−0.0074 (7)	−0.0100 (7)
C12	0.0192 (7)	0.0222 (7)	0.0247 (7)	−0.0009 (5)	0.0002 (5)	−0.0102 (6)
C16	0.0529 (12)	0.0331 (9)	0.0266 (9)	−0.0095 (8)	0.0087 (8)	−0.0101 (7)
C17	0.0410 (10)	0.0318 (9)	0.0244 (8)	−0.0058 (7)	−0.0009 (7)	−0.0128 (7)
C13	0.0326 (9)	0.0262 (8)	0.0256 (8)	−0.0027 (6)	0.0008 (6)	−0.0130 (6)
C14	0.0376 (9)	0.0267 (8)	0.0358 (9)	−0.0067 (7)	0.0010 (7)	−0.0150 (7)
C15	0.0514 (12)	0.0244 (8)	0.0347 (9)	−0.0078 (8)	0.0070 (8)	−0.0084 (7)

### Geometric parameters (Å, º)

**Table:**

Cl1—C4	1.7444 (16)	C10—H10B	0.9800
S1—O2	1.4857 (12)	C10—H10C	0.9800
S1—C1	1.7737 (16)	C11—H11A	0.9800
S1—C12	1.8268 (16)	C11—H11B	0.9800
O1—C7	1.3734 (19)	C11—H11C	0.9800
O1—C8	1.3768 (19)	C12—C17	1.517 (2)
C1—C8	1.355 (2)	C12—C13	1.524 (2)
C1—C2	1.456 (2)	C12—H12	1.0000
C2—C7	1.392 (2)	C16—C15	1.524 (3)
C2—C3	1.403 (2)	C16—C17	1.527 (3)
C3—C4	1.397 (2)	C16—H16A	0.9900
C3—C9	1.501 (2)	C16—H16B	0.9900
C4—C5	1.406 (2)	C17—H17A	0.9900
C5—C6	1.385 (2)	C17—H17B	0.9900
C5—C10	1.506 (2)	C13—C14	1.523 (2)
C6—C7	1.378 (2)	C13—H13A	0.9900
C6—H6	0.9500	C13—H13B	0.9900
C8—C11	1.482 (2)	C14—C15	1.517 (2)
C9—H9A	0.9800	C14—H14A	0.9900
C9—H9B	0.9800	C14—H14B	0.9900
C9—H9C	0.9800	C15—H15A	0.9900
C10—H10A	0.9800	C15—H15B	0.9900
			
O2—S1—C1	108.70 (7)	C8—C11—H11B	109.5
O2—S1—C12	106.89 (7)	H11A—C11—H11B	109.5
C1—S1—C12	98.02 (7)	C8—C11—H11C	109.5
C7—O1—C8	106.43 (12)	H11A—C11—H11C	109.5
C8—C1—C2	107.18 (13)	H11B—C11—H11C	109.5
C8—C1—S1	126.24 (12)	C17—C12—C13	111.82 (13)
C2—C1—S1	126.56 (12)	C17—C12—S1	112.03 (11)
C7—C2—C3	119.57 (14)	C13—C12—S1	107.37 (10)
C7—C2—C1	104.35 (13)	C17—C12—H12	108.5
C3—C2—C1	136.08 (14)	C13—C12—H12	108.5
C4—C3—C2	115.37 (14)	S1—C12—H12	108.5
C4—C3—C9	122.03 (14)	C15—C16—C17	111.07 (15)
C2—C3—C9	122.59 (14)	C15—C16—H16A	109.4
C3—C4—C5	124.84 (15)	C17—C16—H16A	109.4
C3—C4—Cl1	118.17 (13)	C15—C16—H16B	109.4
C5—C4—Cl1	116.99 (12)	C17—C16—H16B	109.4
C6—C5—C4	118.38 (14)	H16A—C16—H16B	108.0
C6—C5—C10	120.13 (16)	C12—C17—C16	110.44 (14)
C4—C5—C10	121.48 (16)	C12—C17—H17A	109.6
C7—C6—C5	117.47 (15)	C16—C17—H17A	109.6
C7—C6—H6	121.3	C12—C17—H17B	109.6
C5—C6—H6	121.3	C16—C17—H17B	109.6
O1—C7—C6	124.62 (15)	H17A—C17—H17B	108.1
O1—C7—C2	111.05 (13)	C14—C13—C12	110.48 (13)
C6—C7—C2	124.33 (15)	C14—C13—H13A	109.6
C1—C8—O1	110.96 (13)	C12—C13—H13A	109.6
C1—C8—C11	134.70 (15)	C14—C13—H13B	109.6
O1—C8—C11	114.33 (14)	C12—C13—H13B	109.6
C3—C9—H9A	109.5	H13A—C13—H13B	108.1
C3—C9—H9B	109.5	C15—C14—C13	111.19 (15)
H9A—C9—H9B	109.5	C15—C14—H14A	109.4
C3—C9—H9C	109.5	C13—C14—H14A	109.4
H9A—C9—H9C	109.5	C15—C14—H14B	109.4
H9B—C9—H9C	109.5	C13—C14—H14B	109.4
C5—C10—H10A	109.5	H14A—C14—H14B	108.0
C5—C10—H10B	109.5	C14—C15—C16	110.65 (14)
H10A—C10—H10B	109.5	C14—C15—H15A	109.5
C5—C10—H10C	109.5	C16—C15—H15A	109.5
H10A—C10—H10C	109.5	C14—C15—H15B	109.5
H10B—C10—H10C	109.5	C16—C15—H15B	109.5
C8—C11—H11A	109.5	H15A—C15—H15B	108.1
			
O2—S1—C1—C8	−8.41 (17)	C5—C6—C7—O1	179.49 (14)
C12—S1—C1—C8	102.54 (15)	C5—C6—C7—C2	−0.4 (2)
O2—S1—C1—C2	169.73 (13)	C3—C2—C7—O1	−177.97 (13)
C12—S1—C1—C2	−79.33 (14)	C1—C2—C7—O1	1.59 (17)
C8—C1—C2—C7	−1.28 (17)	C3—C2—C7—C6	2.0 (2)
S1—C1—C2—C7	−179.71 (11)	C1—C2—C7—C6	−178.49 (15)
C8—C1—C2—C3	178.16 (17)	C2—C1—C8—O1	0.56 (17)
S1—C1—C2—C3	−0.3 (3)	S1—C1—C8—O1	178.99 (11)
C7—C2—C3—C4	−2.1 (2)	C2—C1—C8—C11	−179.83 (17)
C1—C2—C3—C4	178.50 (16)	S1—C1—C8—C11	−1.4 (3)
C7—C2—C3—C9	176.98 (14)	C7—O1—C8—C1	0.42 (17)
C1—C2—C3—C9	−2.4 (3)	C7—O1—C8—C11	−179.28 (13)
C2—C3—C4—C5	1.0 (2)	O2—S1—C12—C17	45.90 (13)
C9—C3—C4—C5	−178.09 (15)	C1—S1—C12—C17	−66.51 (12)
C2—C3—C4—Cl1	−178.93 (11)	O2—S1—C12—C13	−77.25 (12)
C9—C3—C4—Cl1	2.0 (2)	C1—S1—C12—C13	170.35 (11)
C3—C4—C5—C6	0.4 (2)	C13—C12—C17—C16	−55.75 (19)
Cl1—C4—C5—C6	−179.62 (12)	S1—C12—C17—C16	−176.35 (12)
C3—C4—C5—C10	179.79 (15)	C15—C16—C17—C12	56.0 (2)
Cl1—C4—C5—C10	−0.3 (2)	C17—C12—C13—C14	55.82 (18)
C4—C5—C6—C7	−0.7 (2)	S1—C12—C13—C14	179.10 (11)
C10—C5—C6—C7	179.89 (15)	C12—C13—C14—C15	−56.10 (19)
C8—O1—C7—C6	178.78 (15)	C13—C14—C15—C16	56.9 (2)
C8—O1—C7—C2	−1.29 (17)	C17—C16—C15—C14	−56.7 (2)

### Hydrogen-bond geometry (Å, º)

Cg2 is the centroid of the C2–C7 benzene ring.

**Table:**

*D*—H···*A*	*D*—H	H···*A*	*D*···*A*	*D*—H···*A*
C9—H9*A*···O2^i^	0.98	2.53	3.438 (2)	154
C12—H12···O2^i^	1.00	2.39	3.3072 (19)	152
C11—H11b···*Cg*2^i^	0.98	2.83	3.533 (2)	129

Symmetry code: (i) *x*−1, *y*, *z*.
