# Supplementary figures and images for: Crystal structure of 5-chloro-3-cyclo­hexyl­sulfinyl-2,4,6-trimethyl-1-benzo­furan
Source: Acta Crystallogr Sect E Struct Rep Online. 2014 Aug 30;70(Pt 9):o1067–8. doi: 10.1107/S1600536814019217 (PMC4186066; doi:10.1107/S1600536814019217)

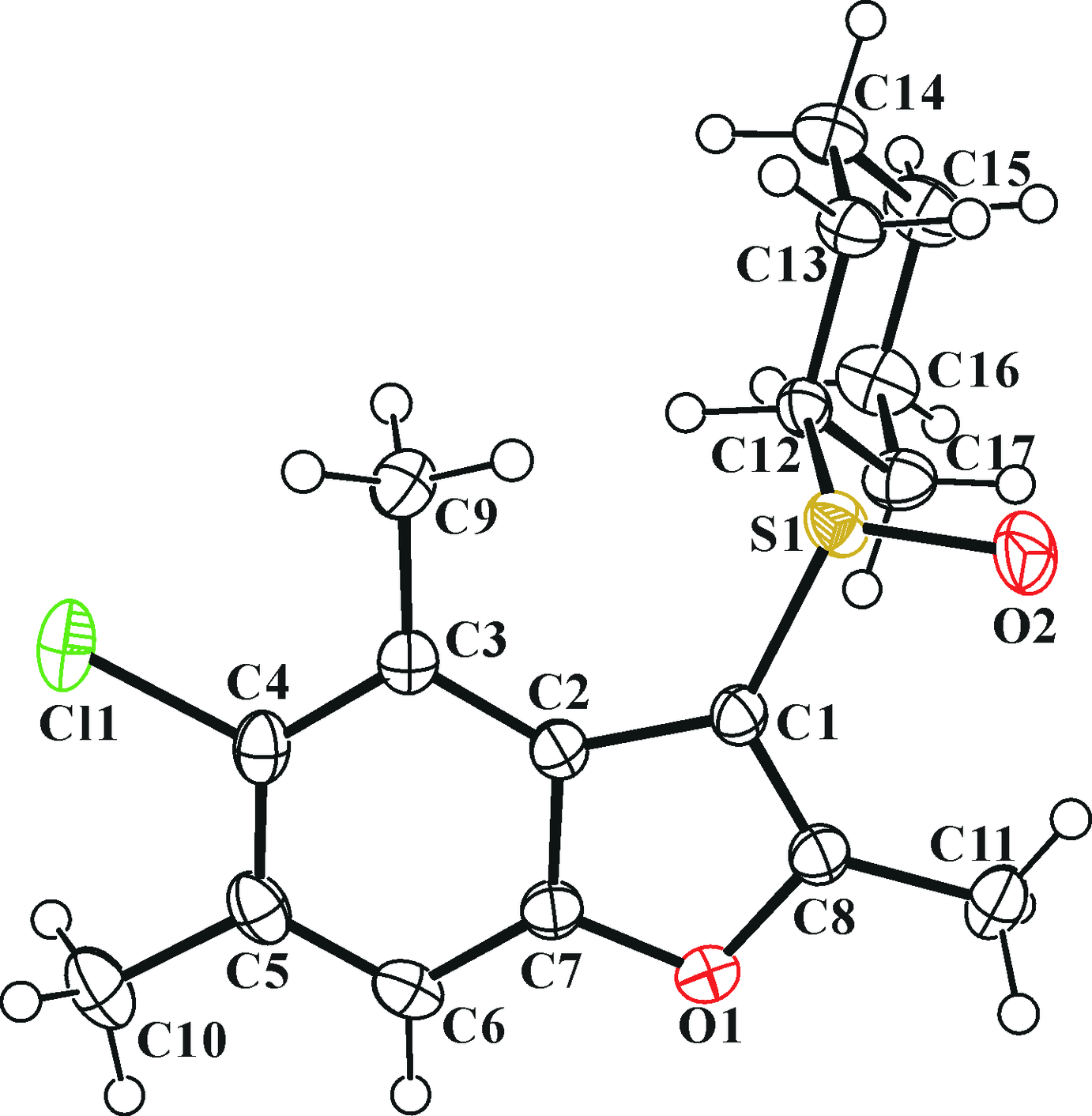

Supplement: Supplementary file 4 [file e-70-o1067-fig1.tif]

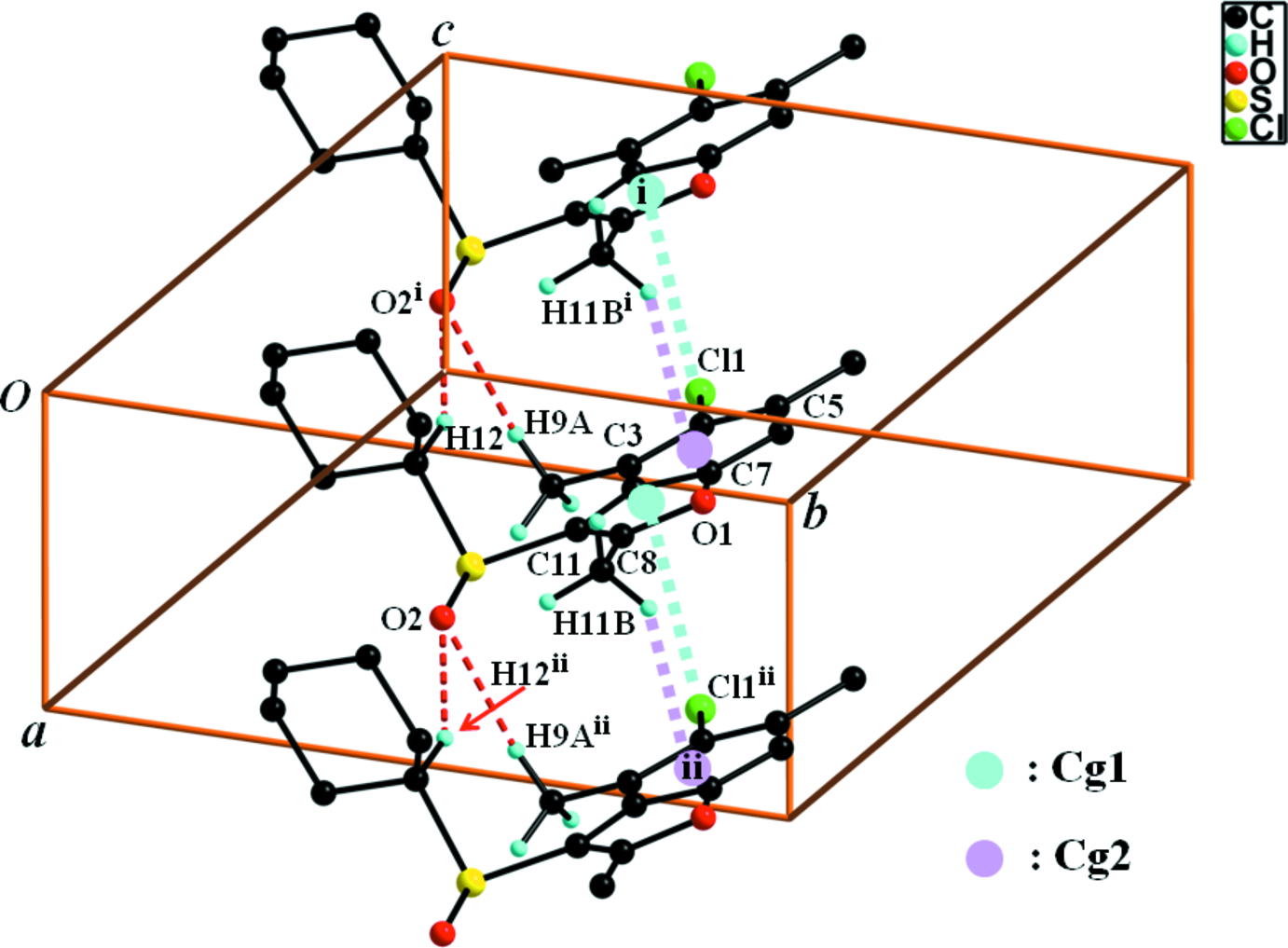

Supplement: Supplementary file 5 [file e-70-o1067-fig2.tif]
